# The Revolving Door Phenomenon in the Romanian Mental Health System

**DOI:** 10.31083/AP38789

**Published:** 2025-02-28

**Authors:** Radu-Mihai Păun, Valentin Petre Matei, Cătălina Tudose

**Affiliations:** ^1^Department of Psychiatry, “Carol Davila” University of Medicine and Pharmacy, 050474 Bucharest, Romania; ^2^“Prof. Dr. Alexandru Obregia” Clinical Psychiatric Hospital, 041914 Bucharest, Romania

**Keywords:** clinical epidemiology, community mental health services, health care

## Abstract

**Background:**

Hospitalized at least three times in a two-year period, have emerged as an unintended side effect of the deinstitutionalization of mental health in high-income countries. Guaranteeing access to high-quality outpatient services has shown to be the most effective method for alleviating the revolving door phenomenon. In Eastern Europe, deinstitutionalization is ongoing, 0 but the phenomenon has received little attention. The present cross-sectional study examined the revolving door phenomenon in the largest psychiatric inpatient unit in Bucharest, Romania.

**Methods:**

Socio-demographic healthcare use and clinical characteristics of 144 patients were collected following admission to the “Profesor Doctor Alexandru Obregia” Psychiatric Hospital via an initial visit conducted between September 2022 and January 2023. A follow-up check occurred one year later to evaluate the number of readmissions and compare those who met the criteria for revolving door status at follow-up with those who did not. After identifying factors associated with revolving door status by univariate analysis, a bivariate model included the results to account for reciprocal moderating effects.

**Results:**

In total, 56 (38.9%) patients met the criteria for revolving door status. The number of lifetime hospitalizations was significantly higher in the revolving door group (odds ratio (OR) = 3.956, *p *≤ 0.001), while involuntary admission on the initial visit decreased the odds of receiving a revolving door status on follow-up (OR = 0.188, *p *= 0.008). Revolving door patients had less time between readmissions than controls (OR = 0.991, *p *< 0.001).

**Conclusions:**

Frequent hospitalization was the primary factor predicting revolving door status in the cohort studied, reflecting the Romanian mental health system’s focus on inpatient care. This illustrates the need for reliable outpatient care as an alternative to hospital admission to avoid the self-perpetuating cycle of repeated admissions that are inefficient both from an economic and medical standpoint.

## Main Points

1. Romania allocates minimal funds to healthcare, focusing primarily on inpatient units, leading to inefficiency. Mental health systems that prioritize outpatient care have fewer inpatient stays, lower readmission rates and reduce overall cost.

2. The revolving door (RD) phenomenon affected 38.9% of the study’s Romanian patient cohort. This prevalence is consistent with global studies, but local factors significantly influence these rates.

3. Patients with a history of frequent hospitalization are more likely to be RD patients. Shorter periods between discharge and readmission were noted. Involuntary admission at baseline showed decreased probability of RD status on follow-up, possibly due to patient reluctance to return to the hospital.

## 1. Introduction

The advent of antipsychotic drugs in the 1950s heralded a paradigm shift in psychiatric care [1]. Following a series of institutional abuse scandals regarding conditions in psychiatric asylums, there was mounting social pressure for change. The reform process, which came to be known as deinstitutionalization, sought to reduce the number of beds in large inpatient units and divert resources to outpatient clinics, community health centers and community based residential facilities. In practice, the closure of long-stay psychiatric hospitals left many former residents with insufficient support [2]. Consequently, this saw the emergence of “revolving door” (RD) patients, defined as patients who are hospitalized at least three times within a two-year period [3, 4].

Deinstitutionalization was largely absent in the Soviet bloc, where institutional care remained the norm until the fall of the Iron Curtain. Although the former communist states have since begun reforming their mental health systems with varying degrees of success [5], there remain major ongoing issues. Most importantly, one is the underfunding and underdevelopment of outpatient services [6]. This often leaves patients, particularly those who cannot afford private healthcare, with no alternatives to the now downsized psychiatric hospitals. The consequent lack of availability of post-discharge services has been identified as a potential cause of the RD phenomenon [7].

Although there are some geographical variations, it is estimated that between a third [8] to a half [9] of psychiatric inpatients are readmitted within one year of discharge. Of pre-discharge predictive factors, the most consistent is the number of previous admissions [10], while other relevant aspects include length of stay, socio-economic status as well as learning disabilities and developmental delays [11]. Post-discharge, general quality of life [12], social isolation [13], non-adherence to treatment, alcohol and substance abuse [11], psychosis and aggression [14] are associated with greater risk of rehospitalization, while good social support and access to quality aftercare services are protective factors [7].

Numerous studies on the predictors of readmission have been conducted, the majority in North American or Western European countries [15]. Comparatively, data from Eastern European countries, where the process of deinstitutionalization has been significantly delayed, is scarce [16]. In this study, clinical and healthcare use were collected in a group of patients admitted to the largest inpatient unit in Romania between September and December 2023 and a follow-up check was conducted one year later to evaluate the number of readmissions and compare those who met the criteria for RD status at follow-up with those who did not. As Romania has one of the lowest healthcare expenditures per capita in Europe, it is important to examine the impact that this has on the quality of mental health services and draw attention to the need for reform.

## 2. Material and Method

### 2.1 Data

The initial cohort comprised 177 individuals selected by simple random sampling from patients admitted to emergency-care wards of the “Prof. Dr. Alexandru Obregia” Clinical Psychiatry Hospital in Bucharest between September 2022 and January 2023. The full details of the initial cohort are described elsewhere [17]. For the present study, patients who were unable to be interviewed due to their cognitive status (e.g., patients with severe dementia or developmental disorders) and patients not residing in Bucharest were excluded, resulting in a study population of 144 patients. The rationale behind the last exclusion criteria was that Romania does not have a centralized digital database that tracks admissions, making the collecting of follow-up data on patients not residing in the hospital’s catchment area difficult and unreliable. A follow-up examination of patient digital records in the hospital database in December 2023, gathered data on the number of readmissions. Revolving-door patients were defined as those who had at least three admissions during the two years prior to follow-up. Patients who met this criterion constituted the study group, while the remainder were designated as controls.

Socio-demographic and clinical data were collected during the baseline visit by interview and from charts and were corroborated by the attending physician and the patient’s family when needed. Diagnoses on admission were grouped in one of the following categories: psychosis (including the International Statistical Classification of Diseases and Related Health Problems 10th Revision (ICD-10) diagnoses of schizophrenia, schizoaffective disorder, schizophreniform disorder and brief psychotic disorder), mania, depression (including both uni- and bipolar depression), substance use disorders and others (any diagnosis that did not belong in any of the previous categories). For patients diagnosed both with substance use disorder and another psychiatric condition, the attending physician was consulted and the one considered the primary reason for admission was chosen.

Disease severity was assessed using the Clinical Global Impression Severity Scale (CGI-S) [18], aggression was evaluated using the modified Overt Aggression Scale (mOAS) [19]. Treatment adherence, defined as taking 80–120% of prescribed psychiatric treatment dose in the four weeks before the current admission (or between the previous hospital discharge and present admission if they were less than four weeks apart), was evaluated only when at least two outside sources were available for corroboration (medical files, family, friends, attending physician, general practitioner).

### 2.2 Statistical Analysis

All analyses were conducted using IBM SPSS Statistics software v26 (Armonk, NY, USA: IBM Corp). Descriptive analysis was used to evaluate social, demographic, and clinical characteristics. Continuous variables were assessed for normality using the Shapiro-Wilk test and analyzed using Mann-Whitney (for non-parametrical distributed data) or the Student *t*-test (for parametrically distributed data). Parametric data are presented with mean and standard deviation, while non-parametric data are given as median and interquartile range. Statistical significance was set at *p *= 0.05. Categorical variables with no more than two categories (e.g., treatment compliance yes/no) were analyzed using Chi-Square while categorical variables with more than two categories (e.g., mean of referral by: police, ambulance, family/friends, self) were individually analyzed using binary logistic regression. Variables that reached statistical significance at first analysis were entered into a multivariate logistic regression.

## 3. Results 

### 3.1 Sociodemographic Characteristics

Sociodemographic characteristics are presented in Table 1. Of the 144 subjects, 38.9% (56) were RD patients. The two groups were similar in age, education, ethnicity, relationship status, housing and employment status. Most RD patients were male (64.3%), single (76.7%), received social benefits (50%) and shared a household with at least one other person (69.6%). Monthly income was lower in the RD group: 1450 ± 1440.9 RON vs 1892.7 ± 1980.5 RON (corresponding to 291 ± 289 EUR vs 381 ± 397 EUR), although the difference was not statistically significant.

**Table 1.** **Socio-demographic characteristics**.

	Non-RD patients (*n *= 88)	RD patients (*n *= 56)	Total (*n *= 144)	Significance (*p-*value, exp(B), 95% CI)
Female Sex	38 (43.1%)	20 (35.7%)	58 (40.2%)	0.390
Age *	45.7 (14.6)	44.6 (15)	45.3 (15)	0.687
Romanian Ethnicity	79 (89.7%)	49 (87.5%)	128 (88.8%)	0.787
Education (Years) *	12.36 (3.7)	11.9 (3.4)	12.2 (3.7)	0.366
Single	55 (62.5%)	43 (76.7%)	98 (68%)	0.099
Living status (living with at least 1 other person)	68 (77.2%)	39 (69.6%)	107 (74.3%)	0.324
Employment status **				
Unemployed (reference category)	25 (28.4%)	15 (26.7%)	40 (27.7%)	Ref.
Employed	28 (31.8%)	13 (23.2%)	41 (28.4%)	0.584; 0.774 (0.309–1.938)
Retired/on social benefits	34 (38.6%)	28 (50%)	62 (43%)	0.445; 1.373 (0.609–3.093)
Average monthly income (EUR)	381 (397)	291 (289)	360 (465)	0.321

*Student *t-*test with results reported as mean (± Standard Deviation (SD)); **Binary logistic regression reported with *p*-value as significance, exp(B) and 95% CI. RD, revolving door; CI, confidence interval.

### 3.2 Healthcare Use Related Factors

Characterization of healthcare use is shown in Table 2. The involuntary admission at the initial visit resulted in a lower probability of RD status on follow-up (exp(B) = 0.392, 95% confidence interval (CI) = 0.155–0.991, *p* = 0.048). Number of mean lifetime admissions prior to baseline was significantly higher (*p* ≤ 0.001) in the RD group (2.67 ± 2.91) than in the control group (0.49 ± 0.83). While the length of the initial hospitalization stay was similar, RD patients had less time between the initial admission and the next hospitalization (142.3 ± 143.6 days vs 324.8 ± 145.1 days for non-RD patients, *p* ≤ 0.001). There were no significant differences in baseline method of admission (e.g., via police, ambulance, family, or self-referral).

**Table 2.** **Factors related to healthcare use**.

		Non-RD patients (*n *= 88)	RD patients (*n *= 56)	Total (*n *= 144)	Significance (*p-*value, exp(B), 95% CI)
Lifetime admissions before initial visit *		0.49 (0.83)	2.67 (2.91)	1.1 (2)	≤ **0.001**
Involuntary status on index visit		48 (54.5%)	20 (35.7%)	68 (47.2%)	**0.040**
Length of initial stay (days)		14.9 (10.93)	14.92 (10.04)	14.9 (10.52)	0.876
Time between initial visit and next admission (days)		142.26 (143.57)	324.8 (145.06)	253.32 (169.47)	≤ **0.001**
Admission method on initial visit **					
	Police (reference category)	19 (21.5%)	11 (19.6%)	30 (20.8%)	Ref.
	Ambulance	17 (19.3%)	13 (23.2%)	30 (20.8%)	0.598; 1.321 (0.469–3.721)
	Family/Friends	29 (32.9%)	17 (30.3%)	46 (31.9%)	0.980; 1.013 (0.390–2.628)
	Referral from medical professional	3 (3.4%)	2 (3.5%)	5 (3.4%)	0.886; 1.152 (0.166–7.990)
	Self-referral	18 (20.4%)	13 (23.2%)	31 (21.5%)	0.674; 1.247 (0.445–3.493)

*Student *t*-test with results reported as mean (± SD); **Binary logistic regression reported with *p* as significance, exp(B) and 95% CI. Bolded values indicate statistical significance.

### 3.3 Clinical Characteristics

The two groups were also similar in clinical characteristics at recruitment admission (Table 3), including diagnosis, disease severity, aggression and treatment compliance. Average disease duration was longer in RD patients (11.2 ± 10 years vs 8.1 ± 10.2 years). The difference was statistically significant in the univariate analysis (*p* = 0.012) but not in the multivariate analysis (Table 4), which controlled for other predictive factors (number of lifetime admissions and involuntary admission on initial visit).

**Table 3.** **Clinical characteristics**.

	Non-RD patients (*n *= 88)	RD patients (*n *= 56)	Total (*n*= 144)	Significance (*p*-value, exp(B), 95% CI)
Diagnosis on initial visit **				
Psychosis (reference category)	34 (38.6%)	22 (39.2%)	56 (38.8%)	Ref.
Mania	11 (12.5%)	9 (16%)	20 (13.8%)	0.656; 1.264 (0.451–3.547)
Depression	23 (26.1%)	13 (23.2%)	36 (25%)	0.760; 0.874 (0.367–2.077)
Substance use disorders	17 (19.3%)	11 (19.6%)	28 (19.4%)	0.999; 1.000 (0.395–2.532)
Others	3 (3.4%)	1 (1.7%)	4 (2.7%)	0.576; 0.515 (0.050–5.273)
Disease duration (Years) *	8.1 (10.2)	11.2 (10)	8.6 (9.9)	**0.012**
Non-compliant to treatment on initial visit	32 (36.3%)	28 (50%)	60 (41.6%)	0.480
Disease severity on initial visit (CGI) *	3.6 (1.4)	3.3 (1.5)	3.5 (1.5)	0.224
Aggression on initial visit (mOAS)*	3.6 (5.6)	2.5 (4.8)	3.6 (5.6)	0.415

*Student *t*-test with results reported as mean (±SD); **Binary logistic regression reported with *p*-value as significance, exp(B) and 95% CI. CGI, Clinical Global Impression; mOAS, modified Overt Aggression Scale. Bolded values indicate statistical significance.

**Table 4.** **Multiple regression analysis of prognostic factors**.

	B	SE	Wald	exp(B)	95% CI	*p*-value
Number of lifetime admissions at initial visit	1.375	0.306	20.158	3.956	2.17–7.212	≤ **0.001**
Disease duration	0.013	0.027	0.238	1.013	0.961–1.069	0.626
Involuntary status at initial visit	–0.1.671	0.628	7.088	0.188	0.055–0.644	**0.008**
Time between initial visit and next admission (days)	–0.009	0.002	2.117	0.991	0.987–0.995	≤ **0.001**

Binary logistic regression reported with *p*-value as significance, exp(B) and 95% CI. Bolded values indicate statistical significance. SE, Standard Error.

### 3.4 Multivariate Analysis

Parameters associated with RD status by univariate analysis were introduced into a binary logistic regression model to account for potential reciprocal moderating effects. Of the four introduced in the model, three maintained statistical significance. Higher numbers of lifetime admissions were predictive of RD status (exp(B) = 3.956, *p* ≤ 0.001), even after controlling for disease duration. Time between readmission after the initial visit remained significantly lower in the RD group (exp(B) = 0.991, *p* ≤ 0.001). Involuntary status at the initial visit significantly decreased the probability of subsequent RD status (exp(B) = 0.188, *p* = 0.008) and the association was greater than in the univariate analysis.

## 4. Discussion

In Romania, as in most other Eastern European countries, the revolving-door phenomenon has received little attention. This is compounded by the fact that per-capita healthcare expenditure in Romania is one of the lowest in Europe [6], with virtually all funds being allocated to inpatient units. This approach is inefficient both from a medical and economic point of view, as mental health systems that prioritize outpatient care see both fewer inpatient stays and lower rates of readmission that lead to lower overall expenditures [20]. By identifying factors associated with RD status in this Romanian patient sample, it is aimed to increase the visibility of this phenomenon and highlight its local particularities.

The percentage of RD patients in the cohort studied (38.9%) is similar to several previous studies (approximately 40%) [21, 22, 23]. A higher prevalence (51%) has been reported by a British study [24], while [25] a lower prevalence (29.3%) was reported in a Chinese sample. Although these variations can be partially attributed to differences in methodology, culture and mental health legislation, it is notable that three papers from the same country [21, 26, 27] had markedly different findings (5.6% vs. 16.7% vs. 40.5%), implying that localized factors play a significant role.

### 4.1 Sociodemographic Factors

In this study, no sociodemographic characteristic was associated with RD status. A potential explanation is that in Romania, while outpatient services are covered by medical insurance are limited to periodic checkups, more complex services such as psychotherapy, behavioral or occupational therapies and addiction centers are only available with payment and others such as halfway homes or mobile crisis units are not available at all. Thus, patients who cannot afford to access more complex aftercare services that might prevent readmission are forced to return to public hospitals. It is worth noting that monthly income in the current cohort was lower than the national minimum wage and less than half of the average national wage (1790 RON ~ 360 Euro vs. 2050 RON ~ 419 Euro vs. 4230 RON ~ 850 Euro). A recent systematic review [15] did not identify any consistent sociodemographic predictors; there is roughly an equal split between studies that found associations between RD and gender, age, ethnicity, education, marital status, employment status, income and urbanicity and those that did not.

### 4.2 Factors Related to Healthcare Use

The number of lifetime admissions before the baseline evaluation was significantly higher for the RD group studied here, even after controlling for disease duration, suggesting that the higher rate at which these patients access hospital care is not simply a function of longer periods of illness. As previous research has established, patients who enter a pattern of frequent hospitalizations tend to maintain it over time [23], further stressing the importance of prevention by ensuring access to high-quality outpatient care. Initial visit length was not predictive of subsequent RD status, although other studies found that shorter admissions were more likely to lead to rehospitalization [28]. The period of time immediately following discharge is known to carry the most risk of readmission [29] and RD patients in the current cohort had much shorter durations between the initial visit and readmission, than non-RD patients. In partial concordance with available data [27, 30, 31], involuntary admission at baseline emerged as a protective factor against RD status. This could be due to the alleviating effect of compulsory hospitalization and treatment on severely ill patients but could also reflect an increased reluctance to access mental healthcare services [28]. Conversely, other authors found a positive association between involuntary admission and RD status, attributing it to the greater severity of psychiatric symptoms in this category of patients [32].

### 4.3 Clinical Factors

Clinical and treatment characteristics, including diagnosis, disease severity, aggression and treatment compliance did not differ between the two groups. Disease duration was associated with RD status in the univariate analysis, but not after controlling for other predictors. While numerous studies cited psychosis and substance use disorders as risk factors [15], there is a significant minority in which there was no link between RD status and psychiatric diagnosis [33, 34]. Diverging from most existing literature, aggression, including self-harm, was not more common in the RD group studied. Nevertheless, this is not unprecedented, as [27] lower levels of lifetime physically violent behavior has been reported for RD patients. As previously speculated by the authors, with regard to sociodemographic variables, this is probably a consequence of the structural characteristics of the Romanian healthcare system, as patients who are not necessarily severely ill but cannot afford to access complex outpatient services after discharge tend to return to public hospitals. This effect appears to be significant enough to mask other variables with smaller effect sizes.

### 4.4 Limitations

The main limitations of this study are the relatively small size of the sample which might not allow for the identification of risk factors with smaller effect sizes and the inclusion of patients only from one hospital’s catchment area, which might affect how representative the study is. However, the source hospital is by far the largest inpatient unit in the country, serving the metropolitan area of a capital city of around three million inhabitants. Potential changes over time in the baseline variables (e.g., losing treatment adherence between hospitalizations) were not accounted for and it was not possible to access information regarding quality of life outside the hospital, social support and access to aftercare services, all of which would have been relevant.

## 5. Conclusions

The main determinant of RD status in the cohort studied was history of hospitalization frequency, irrespective of other factors. Involuntary admission appears to reduce the number of subsequent hospitalizations, but this probably reflects a reluctance by a patient to return to the hospital for fear of going through the same experience with no improvement in their mental health. Other predictors identified in similar studies such as diagnosis, disease severity, aggression or treatment non-compliance were not associated with RD status in the cohort studied here. The most likely explanation is related to the structure of the Romanian mental health system, which concentrates most of its resources in inpatient units, leaving numerous patients, especially those with low socioeconomic status, with no alternative to hospitals for monitoring and treatment. This supports the hypothesis of Oyffe *et al*. [23] that patients who use hospitalization as a shelter against adverse life conditions tend to fall into a cycle of repeated admissions detrimental to both themselves and the efficiency of the healthcare system. This study provides a snapshot of the pitfalls confronted by a mental health system still undergoing deinstitutionalization, particularly of the negative effects of underdeveloped outpatient services.

## Availability of Data and Materials

The data that support this study are available from the corresponding author upon reasonable request.
